# Psychological healing function of poetry appreciation based on educational psychology and aesthetic analysis

**DOI:** 10.3389/fpsyg.2022.950426

**Published:** 2022-09-06

**Authors:** Weijin Zhang

**Affiliations:** School of Humanities, Southeast University, Nanjing, China

**Keywords:** manuscript, poetry appreciation, medical service, mental health, personality shaping, intervention treatment

## Abstract

With the development of society, the rapidly developing social environment has played a significant role in the particular group of college students. College students will inevitably suffer setbacks and psychological obstacles in their studies and daily life. This work aims to ameliorate college students’ various mental illnesses caused by anxiety and confusion during the critical period of status transformation. Educational psychology theory, aesthetic theory, and poetry appreciation are applied to the mental health education of college students to obtain a satisfying psychological healing effect. First, this work summarizes the connotation and characteristics of college student’s mental health and defines educational psychology. Secondly, the long tradition of Chinese poetry teaching is introduced. Besides, the theoretical basis of poetry therapy and aesthetic psychology is expounded, and foreign poetry is discussed. In addition, poetry appreciation is used to promote personality shaping and psychological healing of college students based on the theory of educational psychology and poetry appreciation psychotherapy. In addition, mental health education for college students is studied from the perspectives of psychological health, mental health education, college students’ mental health education, and appreciation of ancient poetry. In addition, the principle and significance of college students’ mental health education are discussed from the perspective of poetry appreciation. Finally, an experimental study is conducted on college students and patients in a specific hospital department by issuing questionnaires to verify the practical application effect of this method in psychotherapy. The survey results indicate that the scores of college students who have completed a one-semester poetry appreciation course in different dimensions of mental disorders are lower than those of those who have not completed the course. At the same time, in the scores of 16 personality traits, the positive trait scores of the experimental group are higher than those of the control group. Comparing scores before and after class also reflects the positive effect of poetry appreciation intervention on college students’ personality shaping. It can be concluded that poetry appreciation has a strong effect on promoting college students’ mental health and personality shaping and improving college students’ psychological problems.

## Introduction

Educational psychology is a scientific study of the basic psychological laws of students, teachers, teaching, and learning and the psychological process of teacher-student interaction in education and teaching context (Meyer and Schutz, 2020; Menz et al., 2021). Educational psychology is closely related to pedagogy (Begeny et al., 2019). Education is a complex and meticulous work of educating people. Education aims to help students develop morally, intellectually, and physically in an all-around way and become social talents. Educational psychology can help teachers discover and master students’ physiological and psychological laws. It provides a psychological basis for clarifying the training objectives of certain educational stages and selecting educational content and methods (Pennycook and Rand, 2021). Educational psychology does not simply interpret the phenomenon of education and teaching with the knowledge of general psychology, nor does it regard education and teaching as the general process of psychological activities. Essentially, it reveals the changes and control laws of the functional system caused by the exchange process and interaction of students’ external and internal information under the influence of education and teaching (Ellemers et al., 2019; Corbett, 2021). Thus, the research object of educational psychology is more extensive and profound than general psychology (Pérez-Ordás et al., 2021).

Today, China is experiencing a social transformation. A cruel but brutal fact is that all social groups are becoming more realistic and eager for quick success and instant benefit. It is well reflected in the vanity and agitation of society (Fossa et al., 2019), giving rise to many social and mental problems (Wise et al., 2019). Some believe that one of the ways to solve these problems is to educate and encourage people to resort to spiritual comfort, namely, healthy, and positive psychology (Ribeiro et al., 2020). As an extraordinary group, college students are in a relatively weak position and need more spiritual care and fit psychological construction (Moreno et al., 2020). In particular, poetry teaching has positively influenced college students’ healthy psychology (Bokhari, 2020). The existing poetry teaching research involves the aesthetic education strategy of poetry language teaching, the comparative study of classical and modern poetry teaching, the comparison of classical and modern poetry teaching, poetry aesthetics, poetry appreciation, poetry teaching, and the transmission of humanistic spirit. However, there is little research on the relationship between poetry teaching and students’ mental health. Therefore, this work utilizes poetry teaching to find effective methods to help vocational middle school students foster healthy psychology (Meagher, 2020).

Based on this, this work first investigates undergraduate patients’ primary psychological status and treatment in an AAA hospital. The specific data are collected through the questionnaire survey of the mental health status of college students. Secondly, a new poetry appreciation-based intervention method is proposed to shape the positive personality of patients to improve their mental health and alleviate mental disorders. This tentative treatment method has particular reference significance for the current psychological intervention treatment system of medical institutions. Therefore, this work provides a reference for the improvement of psychology teaching and contributes to the development of the education industry.

## Overview of the influence of poetry appreciation on mental health

### Research review

Cho and Lee (2021) argued that Chinese ancestors attached great importance to the educational role of poetry and sometimes even put poetry teaching in the primary position of education. In addition, contemporary middle school poetry teaching is also of great significance. Middle school ancient poetry teaching is an integral part of quality education. It is of far-reaching importance in promoting moral education, aesthetic education, and academic education goals in Chinese teaching. However, the current situation of poetry teaching research only stays in the traditional class-type research in the form of reading aloud, appreciation, and exercises (Cho and Lee, 2021). Asrianti et al. (2021) claimed that contemporary education paid much attention to scientific-educational methods and teaching means, and education and teaching should also use reasonable means (educational psychology). Educational psychology has a history of nearly 100 years and studies the psychological process of the interaction between teachers and students and teaching and learning. Properly using psychological knowledge in teaching could increase students’ learning enthusiasm, stimulate learning interest, promote classroom efficiency, and improve students’ learning ability (Asrianti et al., 2021). Hitsuwari and Nomura (2022) reasoned that aesthetics was the characteristic of poetry and poetry teaching. Changing the “rationalism” tendency and the “dismemberment” mode in poetry teaching must retrieve the value orientation and teaching method of aesthetic appreciation. Poetry teaching should become a process of feeling beauty (artistic conception and emotion of poetry). Also, it was a flow of appreciating beauty (content and form of poetry), internalizing beauty (experience and personality of beauty), and creating beauty (thought and action of beauty). Students could master aesthetic knowledge and develop advanced aesthetic abilities (Hitsuwari and Nomura, 2022). Vanzanten (2021) started with the new requirements of the new curriculum reform on classical poetry teaching. He reflected on the current situation of classical poetry teaching in middle schools and analyzed the aesthetic education factors in classical poetry. Both teachers and students should explore and appreciate classical poetry from an aesthetic perspective and feel the artistic charm of classical poetry. In doing so, the classical poetry teaching classroom could be beneficial (Vanzanten, 2021).

To sum up, the current education industry attaches great importance to students’ psychological teaching and aesthetic ability training. However, the current research on poetry appreciation-based psychological healing through psychological teaching and aesthetic analysis is not mature. There is no obvious achievement in this regard. Therefore, the research on the psychological healing function of poetry appreciation based on psychological teaching and aesthetic analysis is innovative.

### Definition of educational psychology and mental health education of college students

Educational psychology is the social psychology to study human learning, the effects of educational interventions, instructional psychology, and school organization in educational contexts (Fisher, 2020). Educational psychology focuses on the application of psychological theories or research findings to education. Educational psychology can be applied to design courses, improve teaching methods, promote learning motivation, and help students face difficulties and challenges in the growth process (Muldoon et al., 2019). Educational psychology and school psychology are often used interchangeably. People who do theoretical work and research are often referred to as educational psychologists, while people who engage in practical work in schools or school-related settings are referred to as school psychologists (Radley et al., 2020). Educational psychology focuses on how students learn and develop. In practice, it pays special attention to students with special educational needs (whether gifted children or children with emotional and behavioral problems). Relationships with other disciplines also contribute to understanding educational psychology. First, educational psychology is based on psychology (Núñez et al., 2019). The research significance of educational psychology is as follows.

(1) The focus of the construction of the teaching staff is teachers, and the quality of teachers includes professional quality and educational quality. Educational psychology is a critical part of educational theory and technology. It helps improve the academic literacy of teachers and the ability to solve practical problems in education.
(2) Educational psychology can help teachers understand students more deeply and improve the pertinence of education and teaching. Studying educational psychology can deepen understanding of the psychological basis of teaching measures to control teaching methods and educational methods actively and scientifically, enrich one’s teaching art, and comprehensively improve teaching quality.
(3) The successful education and teaching reform is supported by educational psychology. Studying educational psychology is conducive to improving the level of dialectical materialism and self-education awareness of teachers, efficiently carrying out the ideological education of students, and improving the scientific nature of teaching and educating people. It is also helpful for teachers to summarize work experience and consciously carry out educational and scientific research.

To sum up, educational psychology is the foundation of current college teaching, so college teaching must improve the education of psychology. Mental health education is the main form and carrier of improving psychological education in colleges and universities. In other words, students’ mental health education is an essential part of the task of teaching and educating people in colleges and universities. College students’ mental health education means that educators teach mental health knowledge in a planned and organized manner according to the characteristics and laws of college students’ psychological development. It aims to help college students establish mental health awareness and master the operation methods, self-regulation, and psychological self-help of mental health. This guidance can promote overall coordination and unity, improve the personality, and influence the mental health development of college students. Building a healthy China requires advocacy of the idea of truth, goodness, and beauty and condensing the positive psychological energy of college students. This work proposes comprehensive educational approaches and countermeasures based on a positive research perspective to effectively maintain and improve the mental health of college students, to achieve their overall physical and psychological development (Ho and Devi, 2020).

### The long tradition of poetry teaching

China is a kingdom of poetry (Lederer et al., 2021). Poetry is the pride and essential part of Chinese literature, recording the joys and sorrows of Chinese people (Fratiwi et al., 2020). Poetry teaching in China has a long tradition (Deng et al., 2020). The so-called “poetry education” originally refers to the “gentle and sincere” educational function in The Book of Songs and later refers to the educational role of poetry in general. For thousands of years, the tradition of teaching Chinese poetry has continued. Conventional poetry teaching has accumulated a lot of successful experience. Poetry has permeated generations (Zhang and Guo, 2021). Poetry carries the moral ideals of Chinese literati, which is a key factor in shaping the personality of Chinese literati. Chinese writer Yu Qiuyu said: “In Europe, the most striking symbol of ancient classics is a world-famous sculpture and buildings that have stood for thousands of years. There have been so many devastating wars in Chinese history that only one kind of classic that is hard to burn is well preserved, ancient poetry and prose classics. These poems are sculptures and architecture embedded in the hearts of countless Chinese, and the transmissivity of reading from generation to generation is the endless corridor of these classics” (Chu, 2020).

The development of foreign poetry probably goes through seven stages: ancient Egypt, Ancient Babylon (BC 40 century to 5th century), the ancient Greek and Roman period (8th century BC to 5th century AD), the Middle Ages (5th to 15th century), also known as the Bible poetry period, the Renaissance period (14th to 16th century), the classical period (17th to 18th century), the Romantic Period (18th to 19th century), and the seventh stage in which aestheticism, symbolism, Imagist surrealism and avant-garde styles emerge in endlessly. The fourth period has great poets such as Britain’s Shakespeare and Italy’s Petrarch. The fifth stage has historical celebrities such as Milton and Voltaire. Romantic poets have bright stars with handsome demeanors, such as Goethe in Germany, Blake and Robert Burns in Britain, the representatives of the Lakeside School like Wordsworth, Coleridge, and Southey, and three young geniuses: Shelley, Byron, and Keats. In the seventh stage, poems of various styles emerge endlessly, and the East and the West have collided, communicated, and fused for nearly a century.

To sum up, whether Chinese or foreign poetry has experienced long-term development and gained excellent sublimation in emotional expression and artistic performance. Most poetry is the leading carrier of expressing the author’s heart condition. Therefore, poetry appreciation through promoting the development of psychological education is a vital breakthrough (Teng et al., 2020).

### Feasibility analysis of poetry teaching in mental health education in colleges and universities

Many students’ behavior and personality disorders are mostly related to their experiences, and psychological factors account for a large proportion. In treating people’s emotional conditions, many people have conducted in-depth discussions throughout the ages (Han et al., 2021). As a unique form of language expression, poetry contains vibrant elements and has a very intuitive and powerful influence on human psychology. Therefore, poetry has become a particular method of psychotherapy. Colleges and universities are essential teaching bases in society. It is fundamental breakthrough research to comprehensively optimize students’ mental health education through poetry to improve students’ mental health.

In addition to medication, recreational therapy is widely used when a person develops a psychological disorder (Zhang et al., 2022). Recreation therapy has a long history and refers to various recreational activities (such as listening to music, learning to sing, watching movies, watching TV, watching dramas, dancing, games, playing chess, playing cards, and visiting gardens). Literature, as an art form, has entertainment functions, which can be seen from its birth. The writing was originally created to meet people’s communication, recreation, and entertainment needs. Chinese poetry or words can be expressed in music. Poetry can be seen as a way of pastime that can disperse mental stress and depression, reduce “sense of bondage,” overcome bad emotions, relax people’s emotions, and positively affect people’s bodies and minds. Poetry and literature can be regarded as a by-product of entertainment to a certain extent, which can also be seen from the identity of the literati. Since poetry is a recreational activity and has the effect of relaxing emotions, it can treat psychological trauma and regulate emotions (Porter et al., 2020). The therapeutic effects of poetry on human emotions are increasingly recognized and used in clinical fields (Plate et al., 2019). Therefore, this paper designs and integrates the analysis of poetry teaching into the mental health teaching of colleges and universities to improve the psychological status of college students through poetry analysis. It can comprehensively promote mental health education and individual development of college education.

### Psychological theory of aesthetic appreciation

Aesthetic appreciation is an advanced form of conscious activity involving perception, emotion, imagination, memory, value evaluation, and other advanced cognitive processes. The birth of aesthetic psychology, an interdisciplinary discipline of aesthetics and psychology in the twentieth century, is the inevitable result of the development and maturity of the two fields. The study of aesthetic psychology initially started from an objective perspective and aimed to confirm the factors affecting aesthetics. It is found that balance, symmetry, and clarity are the main factors affecting aesthetics. At present, the research of aesthetic psychology pays massive attention to the influence of individual differences of aesthetes on aesthetics and supports the subjective construction viewpoint: aesthetics is the aesthetes’ diagnosis and interpretation of artistic works. Aesthetics is a hidden continent, but it is desirable. Although there is no integration theory, researchers have also conducted many experimental studies and constructed some ideas from different angles in recent years (Yang, 2020).

### Necessity of perfecting personality education

Personality education is essential to students’ psychological development. The student period is the golden stage of life because it is a period of physical maturity, psychological transformation, and formation. Life experience and school education at this stage will substantially impact students, some of which are lifelong. Proper personality education for students can promote their formation of good personality qualities to achieve healthy and all-around development. Traditional Chinese education focuses on knowledge and intelligence factors while ignoring the development of students’ non-intelligence factors or personality characteristics. This leads to some students’ weariness, conflict with school, and even personality deviation, which hinders the development of their intelligence and potential. Although personality education pays more attention to the internal spiritual construction of students, it produces less attention to acquiring knowledge and skills. However, this does not mean that personality education and teaching are separated. It improves the learning state from the inside out by cultivating students’ sound personality traits, indirectly promoting acquiring students’ knowledge and skills and developing intelligence (Grosz et al., 2022).

Students’ personality education is the inevitable requirement of social development. From the perspective of social development, schools should cultivate talents needed by the times. Contemporary society is a new society integrating diversity and openness that gives people more opportunities to show and freedom of choice. Under the condition of equal opportunities, individuals are likely to succeed with intelligence and effort. However, there is also the possibility of adventure and failure behind success. Therefore, only individuals who are independent and not afraid of setbacks can be able to face the choices of society and the times and become talents needed by society and the times. Only physically and mentally upright individuals can keep sober and clean in many desires. It can be seen that personality education is vital for contemporary middle school students to form a positive and healthy life attitude and enrich and improve their spiritual realm (Oparina et al., 2020; Cao, 2022).

## Experimental design

Poetry appreciation encourages patients to think independently from their perspective and attitude and comprehensively understand the era and social background of poets based on “writer-centered theory.” Patients can deepen their understanding of the feelings of the times contained in poetry. Excellent poetry often implies patriotism, national righteousness, and noble conduct. While appreciating a poem, patients can empathize with the poem’s emotion and feel its implied connotation and profound significance. At the same time, the positive attitude toward life, exemplary personal character, and indomitable spirit expressed in the poem are examples that patients need to learn to fight against the disease. These spirits play a role in influencing patients’ personality shaping, guiding patients to feel truth and beauty, and distinguishing hypocrisy. Moreover, these spirits help patients establish a correct attitude toward life, set lofty goals, form positive and healthy personality traits, and ultimately improve mental health. The actual effect of poetry appreciation is also restricted by the patient’s understanding ability, the poet’s era, national cultural differences, and other factors. Patients’ interests, attitudes, emotions, values, behavior, and other psychological variables are generally unstable. Therefore, experimental research and descriptive research are combined in the experiment to achieve the research purpose. A short-term longitudinal study is conducted to explore patients’ mental health changes after the poetry appreciation intervention. In addition, the data on the psychological state changes of patients in specific fields before and after the poetry appreciation intervention are collected. This experimental study aims to explain the overall impact of poetry appreciation courses on patients’ mental health and the intervention effect on patients’ short-term psychological state. All the initial data involved are collected through questionnaires or scales and interviews. The experiment does not involve the formal teaching period and the intervention of other teaching contents in the process of investigation and evaluation.

Part of the teaching content (the following poets are well-known ancient Chinese literature figures; poem names are italicized):

1. First, analyze the problems at the first level:

➀ Poems by Li Bai.

Please appreciate this poem (the research object appreciates the work makes discussion)

➁ Ma Zhiyuan’s Poems.

Please appreciate this poem (the research object appreciates the work and makes discussion)

➂ Students practice Dufu’s poems.

Please appreciate this poem (the research object appreciates the work and makes discussion)

2. The second level: “not understanding poetry,” is not understanding the rich meaning of words in poetry or the meaning behind the surface. In other words, students do not know the correspondence between the poet’s choice and arrangement of some keywords and the feelings they convey.

➀ *Bo Chuan Gua Zhou* by Wang Anshi.

Please appreciate this poem (the research object appreciates and makes discussion)

➁Xing Gong by Yuan Zhen.

Please appreciate this poem (the research object appreciates and makes discussion).

3. Appreciation of foreign poetry

➀ Study the poems of Arnold

Please appreciate this poem.

➁ Study of the poems of C. Rossetti

Please appreciate this poem.

➂ Study of the poems of Algerian poets

Please appreciate this poem.

➃ Study of the poetry of the Afghan poet Urfatt

Please appreciate this poem.

Then, the teaching content is analyzed, and in-depth teaching is performed based on students’ experiences and discussion results.

Research object: The research reported here aims to improve the effectiveness of mental health education in colleges and universities by integrating poetry teaching and analysis. Therefore, the research objects are mainly college students. One hundred college students with a stable condition, good basic learning ability, good expression ability, and comprehension ability were randomly selected from a mental disease department of a tertiary hospital as the research object of the questionnaire survey. Among them, 50 patients who received the complete therapeutic intervention for poetry appreciation served as the experimental group. The experimental group was given a single intervention for 15 days, and the intervention results were comprehensively analyzed and evaluated. Another 50 patients who did not receive poetry appreciation therapy were selected as a control group. Then, the two groups of students were given mental health education, and the experimental group was given poetry teaching and appreciation intervention. Before and after the intervention, a questionnaire survey was conducted on the patients in the two groups to analyze the changes in the mental health and personality characteristics of the patients in different groups. The research object does not involve teaching content such as school education; there is no intervention of other factors in the research process. Table 1 lists the specific information of the study subjects.

**TABLE 1:** Specific information about the research object.

Grouping	Gender	Number of people	Total
Control group	Male	26	50
	Female	24	
Experimental group	Male	23	50
	Female	27	

Experimental tools include a basic personal information questionnaire, literary aesthetic questionnaire—academic subscale, Symptom Checklist 90 (SCL-90), and Sixteen Personality factors questionnaire (16PF).

Specifically, the literary aesthetic questionnaire, compiled by Chinese scholar (He et al., 2020) is a nationally recognized literary aesthetic evaluation scale. SCL-90 is a self-assessment scale of mental health widely used worldwide, including nine influencing factors, such as somatization, obsessive-compulsive disorder, and anxiety. It is often used to evaluate the symptoms of mental disorders. 16PF is a personality measurement tool, including 16 typical personality characteristics, such as warmth, reasoning, emotional stability, and sensitivity; it has high recognition, reliability, and validity worldwide. Table 2 shows the critical contents of this paper.

**TABLE 2:** Important research contents of this experiment.

Question	Question content (1–5 indicate very serious, serious, not serious, very not serious)
Q1	Have you been taught psychology?
Q2	Are you taught aesthetic analysis?
Q3	Have you been taught poetry appreciation?
Q4	Ask about your current obsessive-compulsive disorder performance score.
Q5	Please rate your current relationship status.
Q6	Please rate your current depression status.
Q7	Please rate your current anxiety disorder status.
Q8	Please rate your current state of paranoia.
Q9	Please rate your current state of hostility.
Q10	Please rate your current mental illness status.
…	

Data analysis: IBM SPSS 25.0 statistical software is chosen to analyze the statistical data. The scores of each dimension of the influencing factors of patients’ mental health and personality traits are recorded as the mean and standard deviation in the questionnaire. Then, the chi-square test and Analysis of Variance (ANOVA) are performed for a single factor. The calculation of the chi-square test and variance analysis reads:


(1) $$\chi^{2}=\sum_{i=1}^{k}\frac{{\left(f_{i}-n\,p_{i}\right)}^{2}}{n\,p_{i}}$$

(2) $$D\,X=\sum_{i=1}^{n}{\left(x_{i}-E\,X\right)}^{2}\,p_{i}$$

Through these two analysis methods, this work improves the total evaluation value of the research results, thus improving the research value.

## Influence of poetry appreciation intervention on the mental health and personality traits of patients

### Influence of poetry appreciation intervention on the mental health of patients

**FIGURE 1:**
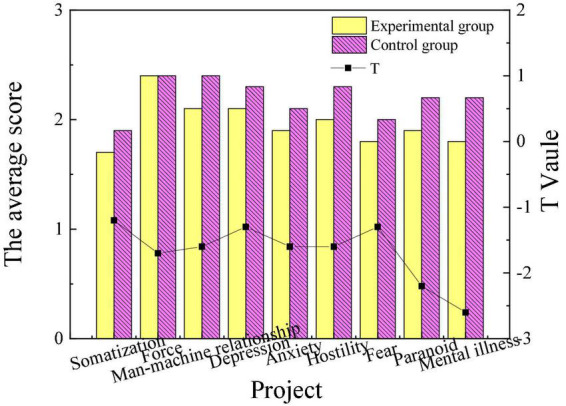
Comparison of mental health of the experimental group and the control group before intervention.

**FIGURE 2:**
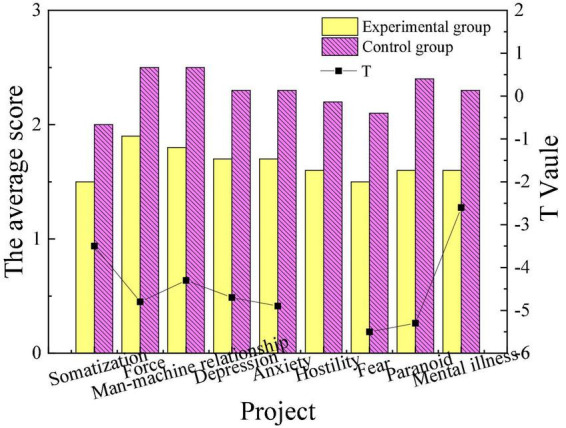
Comparison of mental health of the experimental group and the control group after intervention.

This work mainly combines educational psychology theory and aesthetic theory with poetry appreciation and intends to apply it to college students’ mental health education. Relevant investigation and research are carried out to verify the psychotherapy effect of this method. The scores of mental health of patients in the experimental group and the control group before and after intervention treatment are shown in Figures 1, 2.

From Figure 1, there are no apparent differences in the mental health levels of the patients in the experimental group and the control group before the intervention treatment. After the intervention treatment, the scores of the patients in the experimental group who completed the intervention treatment of poetry appreciation had a significantly lower score in obsessive-compulsive, interpersonal relationship, depression, anxiety, paranoia, hostility, and mental illness than those in the control group (*P* < 0.05). This indicates that the overall mental health level of the experimental group is higher than that of the control group. The result suggests that the intervention treatment of poetry appreciation plays a positive role in the mental health of the students in the experimental group, and has obvious remission and adjustment effects on symptoms such as mental disorders.

### Influence of poetry appreciation intervention on the personality trait of patients

**FIGURE 3:**
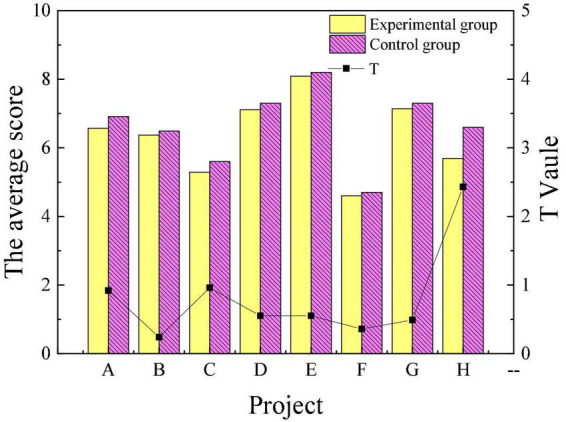
Comparison of personality traits of the experimental group and the control group before intervention (A–H).

**FIGURE 4:**
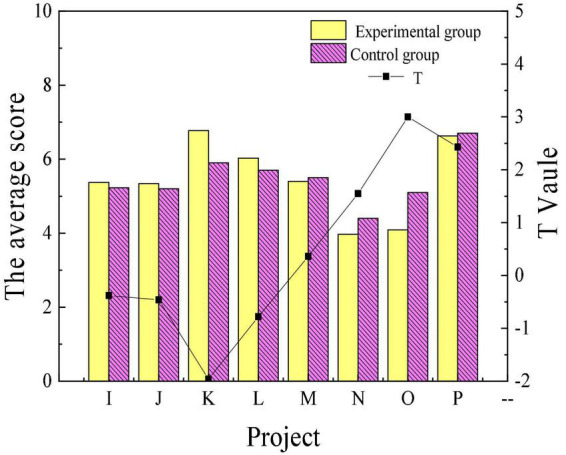
Comparison of personality traits of the experimental group and the control group before intervention (I–P).

Figures 3, 4 reveal the scores of personality traits through the 16PF before the intervention treatment of poetry appreciation. Items A to P represent warmth, reasoning, emotional stability, dominance, liveliness, rule consciousness, social boldness, sensitivity, vigilance, abstractedness, privateness, apprehension, openness to change, self-reliance, perfectionism and tension, respectively.

**FIGURE 5:**
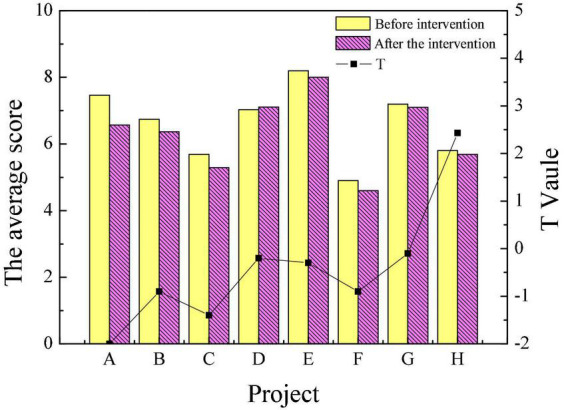
Comparison of personality traits of the experimental group before and after intervention (A–H).

**FIGURE 6:**
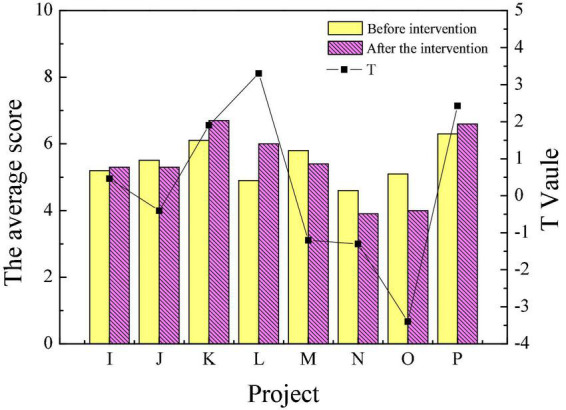
Comparison of personality traits of the experimental group before and after intervention (I–P).

Figures 3, 4 suggest that there were only significant differences in the sensitivity and perfectionism scores of the experimental and control groups in 16 personality traits before the intervention. Overall, the personality traits of patients in the experimental group and the control group before the experiment are similar. Figures 5, 6 reveal the comparison of personality traits of patients in the experimental group before and after the intervention treatment of poetry appreciation.

The results presented in Figure 5, 6 show that the scores of the experimental group in dominance, vigilance, tension, apprehension, and privateness decrease after the intervention treatment of poetry appreciation. Meanwhile, the experimental group’s scores in other traits such as warmth, reasoning, and emotional stability increased after the intervention. There are significant differences in the scores of warmth, privateness, apprehension, and perfectionism (*P* < 0.05). Tables 3, 4 summarizes the test status of the significance of psychological changes for students of different genders and groups.

**TABLE 3:** Psychological changes of different genders.

	A	B	C	D	E	F	G	H
Male	0.59	0.42	0.76	0.66	0.7	0.47	0.53	0.58
Female	0.81	0.61	0.58	0.71	0.52	0.61	0.57	0.67
	
	**I**	**J**	**K**	**L**	**M**	**N**	**O**	**P**
	
Male	0.71	0.53	0.65	0.73	0.67	0.74	0.58	0.69
Female	0.69	0.61	0.42	0.74	0.46	0.44	0.44	0.59

**TABLE 4:** Changes in the experimental group and control group.

	A	B	C	D	E	F	G	H
Experimental group	0.59	0.42	0.76	0.66	0.7	0.47	0.53	0.58
Control group	0.04	0.04	0.02	0.02	0.04	0.04	0.04	0.04
	
	**I**	**J**	**K**	**L**	**M**	**N**	**O**	**P**
	
Experimental group	0.71	0.53	0.65	0.73	0.67	0.74	0.58	0.69
Control group	0.03	0.03	0.02	0.02	0.01	0.01	0.04	0.05

Tables 3, 4 indicate that the intervention treatment of poetry appreciation has a positive effect on the personality shaping of patients, especially showing an obvious promotion of the positive factors (warmth and perfectionism) and significant amelioration of negative factors (privateness and apprehension). The results indicate that intervention treatment of poetry appreciation has a considerable mitigation effect on negative personality and effective promotion of the pursuit of self-worth and self-restraint of patients.

Tables 5, 6 summarize the F-test results of the psychological scores of the observation objects of different genders and groups.

**TABLE 5:** Psychological differences of different gender students.

Indicator	Female	Male
Average value	0.625625	0.591875
Variance	0.010186	0.01327
Observed value	16	16
Df	15	15
*F*	0.767639	0
*P* (*F* ≤ f) Single-tailed test	0.307517	0
*F* Single-tailed critical value	0.416069	0

**TABLE 6:** Psychological differences among different groups of students.

Indicator	Experimental group	Control group
Average value	0.625625	0.030625
Variance	0.010186	0.000153
Observed value	16	16
Df	15	15
F	66.61308	0
*P* (*F* ≤ f) Single-tailed test	0	0
*F* Single-tailed critical value	2.403447	0

Tables 5, 6 suggest that in the analysis of psychological differences of students of different genders, the corresponding variance values are small, all less than 0.02. It can be considered that there is no significant difference. In contrast, there is a large difference in the value of psychological change. The result demonstrates that there is a significant difference in the value of psychological change between the two groups.

## Discussion

Reference (Vanzanten, 2021) starts from the new requirements of the new curriculum reform for the teaching of classical poetry, reflects on the current situation of the teaching of classical poetry in middle schools, and analyzes the factors of aesthetic education in classical poetry. Vanzanten believes that both teachers and students should explore and appreciate classical poetry from the aesthetic point of view and feel the artistic charm of classical poetry. It can be seen that scholars believe that the power of beauty contained in classical poetry can have a positive impact on students. On the basis of the previous research, this paper innovatively applies the educational psychology theory, aesthetic theory, and poetry appreciation to the mental health education of college students, hoping to obtain a satisfactory psychotherapy effect. Experiments suggest that poetry appreciation can indeed play an auxiliary role in the treatment of students with psychological problems. This work mainly explores the psychological problems of college students through poetry appreciation and analysis and provides a reference for solving the psychological problems of college students. The research aims to comprehensively promote the far-reaching development of college education work based on improving the teaching effect of colleges and universities. According to the questionnaire survey, there was no significant difference in the mental health level of the experimental and control groups before the intervention. After the intervention treatment, the scores of the patients in the experimental group who completed the intervention treatment of poetry appreciation were significantly lower than those in the control group in terms of obsessive-compulsive disorder, interpersonal relationship, depression, anxiety, paranoia, hostility, and mental illness (*P* < 0.05). The results indicate that the overall mental health level of the experimental group is higher than that of the control group. The intervention treatment of poetry appreciation has a positive effect on the mental health of the students in the experimental group and has a significant impact in relieving and adjusting symptoms such as psychological disorders. Second, before the intervention, there were only significant differences between the test and control groups in sensitivity and perfectionism scores for 16 personality traits. Overall, the test patients’ personality traits were similar to those in the control group before the experiment. Finally, after the poetry appreciation intervention, the experimental group’s dominance, vigilance, tension, fear, and privacy scores decreased. At the same time, the scores of other traits such as warmth, reasoning, and emotional stability increased in the post-intervention experimental group, while there were significant differences in the scores of warmth, intimacy, worry, and perfectionism (*P* < 0.05). Therefore, the intervention treatment of poetry appreciation has a positive effect on the patient’s personality shaping, especially the positive factors (enthusiasm, perfectionism) are significantly promoted, and the negative factors (privacy and anxiety) are significantly improved. The results suggest that the intervention treatment of poetry appreciation significantly relieves negative personality and effectively promotes patients’ pursuit of self-worth and self-restraint. It can be seen that poetry appreciation can effectively relieve the anxiety of college students, improve their enthusiasm for learning, effectively improve the psychological problems of college students, and promote the comprehensive development of college teaching.

## Conclusion

This work explores the practical application effect of poetry appreciation based on educational psychology and aesthetic analysis in psychotherapy through questionnaires to improve the quality of college students’ mental health education. It is found that the appreciation of excellent poetry has a certain soothing effect on the mental disorders of patients and has a certain promoting effect on the shaping of positive psychological factors. At the same time, it can improve the positive personality traits of patients, improve the negative factors in the personality traits, and open up a new way for medical institutions to treat patients with mental disorders. The most prominent manifestation of the breadth and depth of Chinese culture lies in the many classic Chinese poems worthy of inheritance. As outstanding contemporary Chinese citizens, college students should improve their cognitive and mental health levels in poetry appreciation and establish good moral cultivation and personality traits through a positive attitude towards life. Only in this way can students become the inheritors of Chinese culture and the successors of the socialist cause. However, there are still some flaws to be improved. Poetry appreciation requires long-term training to expand its positive impact. It was found that the results of different gender groups and experimental groups showed significant changes after the intervention through the intervention. It can be seen that the research reported here can fully improve the psychological status of students and play a critical role in the development of college education.

However, although this work provides a relatively complete reference method for the research on psychological problems of college students and studies its practical application, it is not perfect for applying and promoting this method. Therefore, future research will expand the research on poetry appreciation in improving students’ psychological problems to promote educational development.

## Data availability statement

The raw data supporting the conclusions of this article will be made available by the authors, without undue reservation.

## Ethics statement

The studies involving human participants were reviewed and approved by the Southeast University Ethics Committee. The patients/participants provided their written informed consent to participate in this study. Written informed consent was obtained from the individual(s) for the publication of any potentially identifiable images or data included in this article.

## Author contributions

The author confirms being the sole contributor of this work and has approved it for publication.
